# Angiotensin II receptor inhibition ameliorates liver fibrosis and enhances hepatocellular carcinoma infiltration by effector T cells

**DOI:** 10.1073/pnas.2300706120

**Published:** 2023-05-01

**Authors:** Li Gu, Yahui Zhu, Maiya Lee, Albert Nguyen, Nicolas T. Ryujin, Jian Yu Huang, Shusil K. Pandit, Shadi Chamseddine, Lianchun Xiao, Yehia I. Mohamed, Ahmed O. Kaseb, Michael Karin, Shabnam Shalapour

**Affiliations:** ^a^Laboratory of Gene Regulation and Signal Transduction, Department of Pharmacology and Pathology, School of Medicine, University of California San Diego, San Diego, CA 92093; ^b^Department of Center of Smart Laboratory and Molecular Medicine, School of Medicine, Chongqing University, Chongqing 400044, China; ^c^Department of Cancer Biology, The University of Texas MD Anderson Cancer Center, Houston, TX 77030; ^d^Gastrointestinal Medical Oncology Department, The University of Texas MD Anderson Cancer Center, Houston, TX 77030; ^e^Department of Biostatistics, The University of Texas MD Anderson Cancer Center, Houston, TX 77030

**Keywords:** NASH-driven HCC, anti-PD-1, losartan, liver fibrosis

## Abstract

Although viral hepatocellular carcinoma (HCC) is declining, nonviral HCC, which often is the end stage of nonalcoholic or alcoholic steatohepatitis (NASH, ASH), is on an upward trajectory. Immune checkpoint inhibitors (ICIs) that block the T cell inhibitory receptor PD-1 were approved for treatment of all HCC types. However, only a minority of HCC patients show a robust and sustained response to PD-1 blockade, calling for improved understanding of factors that negatively impact response rate and duration and the discovery of new adjuvant treatments that enhance ICI responsiveness. Using a mouse model of NASH-driven HCC, we identified peritumoral fibrosis as a potential obstacle to T cell–mediated tumor regression and postulated that antifibrotic medications may increase ICI responsiveness. We now show that the angiotensin II receptor inhibitor losartan, a commonly prescribed and safe antihypertensive drug, reduced liver and peritumoral fibrosis and substantially enhanced anti-PD-1-induced tumor regression. Although losartan did not potentiate T cell reinvigoration, it substantially enhanced HCC infiltration by effector CD8^+^ T cells compared to PD-1 blockade alone. The beneficial effects of losartan correlated with blunted TGF-β receptor signaling, reduced collagen deposition, and depletion of immunosuppressive fibroblasts.

## Significance

Immune checkpoint inhibitors are used in HCC treatment, but overall response rates for single-agent PD-1/PD-L1 blockers have remained stubbornly low. Using a mouse model of NASH-driven HCC, we show that cotreatment with the safe and inexpensive angiotensin II receptor inhibitor losartan substantially enhanced anti-PD-1-triggered HCC regression. Although losartan did not potentiate the reinvigoration of exhausted CD8^+^ T cells, it considerably enhanced their intratumoral invasion, which we postulated to be compromised by peritumoral fibrosis. Indeed, the beneficial effect of losartan correlated with inhibition of TGF-β signaling and collagen deposition, and depletion of immunosuppressive fibroblasts. Losartan should be evaluated for its adjuvant activity in HCC patients undergoing PD-1/PD-L1 blocking therapy.

Hepatocellular carcinoma (HCC), one of the most common cancer types worldwide (1), is the end result of chronic liver injury and inflammation, often occurring in the context of hepatocyte cell death and liver fibrosis (2, 3). Whereas early and locoregional HCC are effectively treated by surgical resection, radiofrequency ablation, or chemoembolization, the treatment of advanced HCC is limited by the compromised liver function accompanying the disease (1). The only approved targeted HCC therapies are pan-kinase inhibitors, such as sorafenib, which extend patient survival by several months, leaving the 5-y survival rates at 30% for patients with localized disease and an abysmal 2.5% for patients with advanced metastatic disease (4, 5). A considerable advance in HCC treatment was the finding that immune checkpoint inhibitors (ICIs) targeting PD-1 or its ligand PD-L1 [hereafter PD-(L)1], whose association causes T cell exhaustion (6), can curtail HCC growth or induce tumor regression with objective response rates (ORRs) of 15 to 20% (7–11). Although the effectiveness of PD-(L)1 blockade was recently improved by its combination with Vascular endothelial growth factor (VEGF) receptor inhibitors or CTLA4 checkpoint blockade (11), ORRs remain lower than 30% and suggested to be particularly low in nonalcoholic steatohepatitis (NASH)-related HCC (12–14). Although hepatosteatosis was postulated to account for the adverse effect of NASH on ICI responsiveness (12–14), it should be recognized that hepatosteatosis usually declines or disappears (“burnout NASH”) in advanced NASH, which is characterized by extensive fibrosis, which often precedes HCC (15). Moreover, hepatosteatosis is not required for induction of liver damage and fibrosis in NASH-afflicted mice (16). Based on our studies of NASH-related HCC in the *MUP-uPA* mouse model (17–19), we reasoned that liver fibrosis is more likely to account for the adverse effect of NASH on ICI outcome than hepatosteatosis. Although NASH is driven by metabolic inflammation, it is also accompanied by marked changes in the hepatic immune system, including the accumulation of immunosuppressive IgA-expressing plasma cells, which dismantle immunosurveillance by HCC-directed T cells (19, 20). The immunosuppressive activity of IgA^+^ plasma cells is IL-10 and PD-L1 dependent, and either PD-L1 or IL-10 blockade, or ablation, restore anti-HCC immunity to high fat diet (HFD)-fed *MUP-uPA* mice (19), which develop NASH and robustly progress to HCC (17, 18). While most NASH-induced liver tumors in *MUP-uPA* mice were effectively eliminated by PD-L1 blockade, tumors with extensive peritumoral fibrosis were treatment refractory (19).

Liver fibrosis or excessive collagen fiber deposition is triggered by chronic liver injury, which induces production of transforming growth factor-beta (TGF-β) and other profibrogenic cytokines by activated immune cells, mainly macrophages (21, 22). TGF-β activates collagen-producing hepatic stellate cells (HSC) that express α-smooth muscle actin (α-SMA) and glial fibrillary acidic protein (23) and gives rise to cancer-associated fibroblasts (CAF) during HCC emergence (24, 25). TGF-β activates HSC by binding to its type II receptor (TGFBR2) which heterodimerizes with the type I receptor (TGFBR1), triggering activation and nuclear translocation of SMAD2, 3, and 4 transcription factors (26). TGFBR signaling is potentiated by angiotensin II (Ang II) acting via its type 1 receptor (AngIIR1) (27, 28). Although no TGFBR inhibitors or other targeted therapeutics were approved for the treatment of liver fibrosis (29), the commonly used AngIIR1 inhibitor and antihypertensive drug losartan can reduce liver fibrosis in humans (30, 31) and rodents (32, 33). Other studies carried out by Rakesh Jain’s group have shown that losartan enhances anticancer drug delivery (34) and down-regulates immunosuppression-associated genes in ovarian and pancreatic cancers when combined with chemo- or radio-therapy (35, 36) and reduced ICI-induced edema in glioblastoma mouse model (37). Inspired by these findings, we investigated whether losartan improves ICI-induced HCC regression and if so, whether this correlates with its antifibrogenic activity. We now show that losartan potentiates the therapeutic response to a suboptimal PD-1 antagonistic antibody in the *MUP-uPA* model and that this effect correlates with improved intratumoral invasion by reinvigorated CD8^+^ cytotoxic T cells, diminished collagen type I (Col I) production, and down-regulated TGF-β signaling.

## Results

### Losartan Potentiates Anti-PD-1-Induced HCC Regression.

**Fig. 1.**
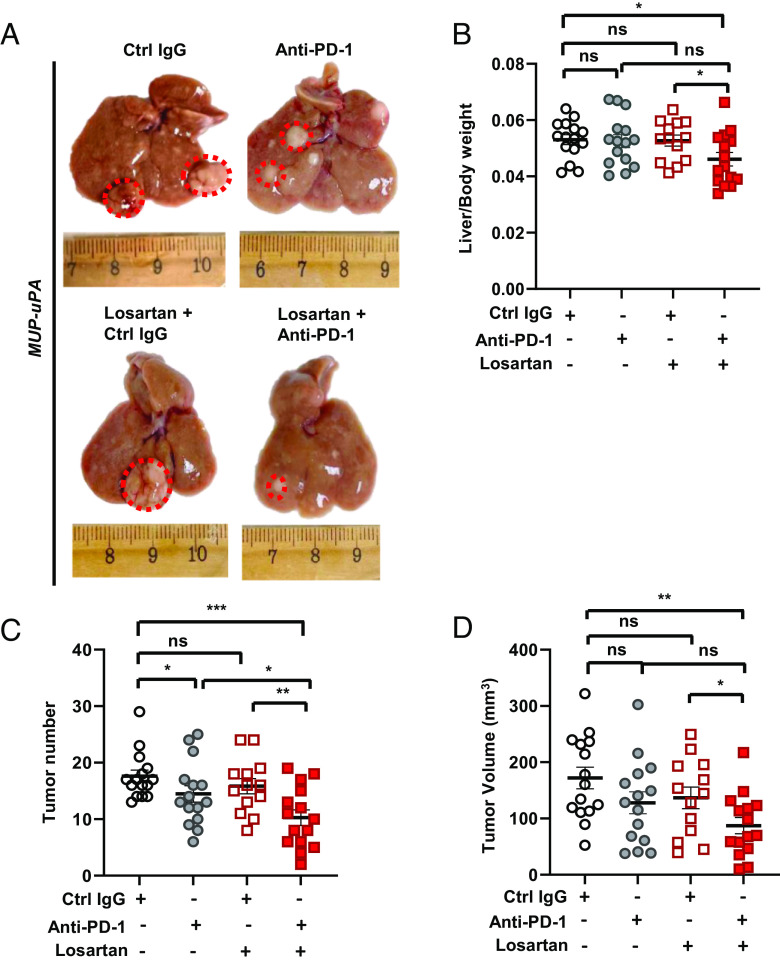
Losartan potentiates anti-PD-1-induced regression of NASH-driven HCC. (*A*) Gross liver morphology in HFD-fed *MUP-uPA* mice that were treated with ctrl IgG, anti-PD-1, losartan+ctrl IgG, and losartan+anti-PD-1 (n = 13 to 15). (*B*) Liver/body weight ratio in the above mice at the end of the treatments. (*C* and *D*) Tumor multiplicity (*C*) and volume (*D*) at the end of treatments. Data are presented as mean ± SEM. *P < 0.05, **P < 0.01, ***P < 0.001, ns, not significant (unpaired two-tailed *t* test and Mann–Whitney *U* test were used to determine significance).

NASH-driven HCC in *MUP-uPA* mice was the model chosen for the present study as its pathogenic mechanisms, transcriptome, and mutational signature resemble human HCC and are also responsive to PD-(L)1 blockade (17, 19). We first determined the optimal losartan dose and treatment regimen. We found that losartan in drinking water was well tolerated at 30 mg/kg, with no significant weight loss when the mice were treated for ~2 mo (*SI Appendix,* Fig. S1 *A* and *B*). However, when the treatment period exceeded 2 mo and the dose was raised to 50 mg/kg, HFD-fed mice no longer gained weight and a tendency to develop smaller tumors (*SI Appendix,* Fig. S1 *A–D*). We next examined whether losartan can potentiate anti-PD-1-induced HCC regression. We placed 6-wk-old *MUP-uPA* mice on HFD for 6 mo to induce NASH and HCC and allocated HCC-bearing mice into four treatment groups (*SI Appendix,* Fig. S1*E*): control (ctrl) IgG, anti-PD-1, losartan+ctrl IgG, and losartan+anti-PD-1. Treatments lasted 8 wk while the mice were kept on HFD. Under these conditions, body weight gain was identical across all groups and no organ injury was observed (*SI Appendix*, Fig. S1 *F* and *G*). Notably, the combination of losartan with anti-PD-1 resulted in lower liver/body weight ratio, tumor multiplicity, and tumor volume compared to ctrl IgG, anti-PD-1 alone, or ctrl IgG plus losartan (Fig. 1 *A–D*). Importantly, losartan addition augmented anti-PD-1-induced tumor regression. However, anti-PD-1 single treatment also caused a moderate decrease in hepatosteatosis, liver triglyceride (TG) accumulation, and serum TG amounts, effects that were slightly affected by losartan addition (*SI Appendix,* Fig. S1 *H*–*J*). Losartan without or with anti-PD-1 reduced liver damage marked by the presence of liver enzymes in the circulation (*SI Appendix,* Fig. S1*K*).

### Losartan Enhances HCC Infiltration with Anti-PD-1-Induced T Effector Cells.

**Fig. 2.**
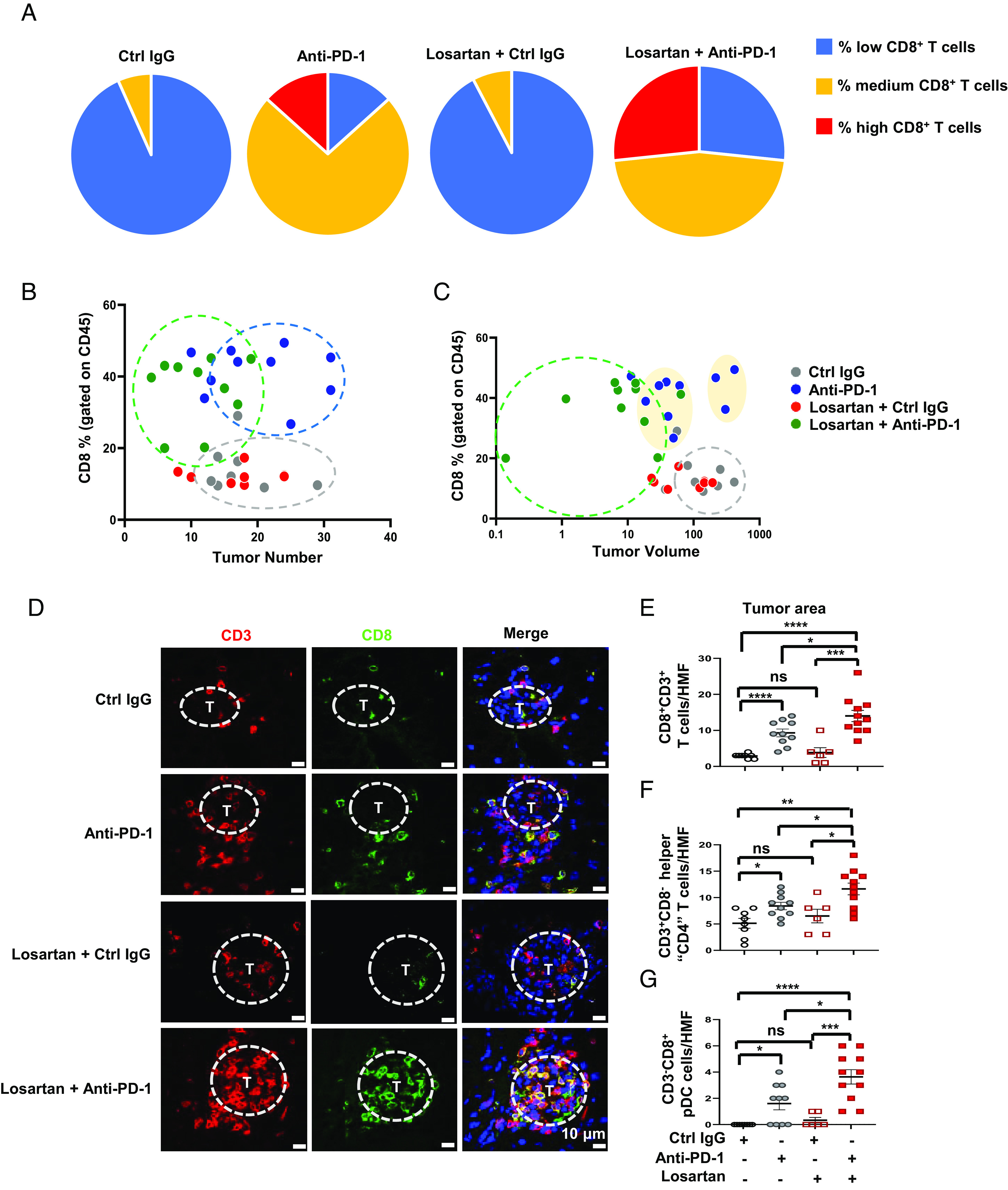
Losartan stimulates intratumoral infiltration by reinvigorated Teff cells. (*A*) *MUP-uPA* mice from each group in Fig. 1*A* were classified into three categories based on the number of CD8^+^ T cells in their livers (low < 1 million; medium 1 to 4.6 million; high > 4.6 million). The percentages of mice in each category are indicated (n = 13 to 15). (*B* and *C*) Correlation between liver CD8^+^ T cell frequency and treatment outcome determined as either tumor multiplicity (*B*) or tumor volume (*C*). (*D–G*) Frozen liver sections were stained for CD3 and CD8 and counterstained with DAPI. T-tumor. Scale bars, 10 µm (*D*). Quantification of CD8^+^CD3^+^ T cells (*E*), CD3^+^CD8^-^ helper “CD4” T cells (*F*), and CD3^−^CD8^+^ pDC cells (*G*) into tumors is based on cell quantitation per high-magnification field (HMF) in tumor and nontumor areas by Image J analysis of 10 fields of per section. Data are presented as mean ± SEM. *P < 0.05, **P < 0.01, ***P < 0.001, ****P < 0.0001, ns, not significant (unpaired two-tailed *t* test and Mann–Whitney *U* test).

To identify how losartan enhanced anti-PD-1-induced HCC regression, immune cell numbers and effector functions were analyzed by flow cytometry (FC). Both CD8^+^ T cell number and fraction were increased in the livers of anti-PD-1-treated mice, with the majority of CD8^+^ T cells secreting TNF and IFNγ (Fig. 2*A* and *SI Appendix,* Fig. S2 *A*–*C*). Additionally, there was a decline in the percentage of CD8^+^ T cells that express the inhibitory collagen receptor LAIR1 (38) after anti-PD-1 treatment; however, the LAIR1 expressing in liver monocytes barely changed (*SI Appendix,* Fig. S2 *D* and *E*). Losartan had no effect on total CD8^+^ T cell number, their effector function, or expression of LAIR1 (*SI Appendix,* Fig. S2 *A*–*E*). Nonetheless, the combination of losartan with anti-PD-1 increased the number and percentage of CD8^+^ T cells associated with lower tumor number and volume in comparison to anti-PD-1 alone (Fig. 2 *A*–*C*). Anti-PD-1 without or with losartan enhanced the expression of mRNAs coding for Ccl8, Ccl5, Ccl19, Ccl2, Cxcl9, and Cxcl10, which are chemokines that promote T cell recruitment and activation (*SI Appendix,* Fig. S2 *F*–*H*). Losartan cotreatment tended to further increase chemokine expression, but the effect was not statistically significant. FC and immunochemistry (IHC) revealed that while losartan in combination with anti-PD-1 did not exert a significant effect on anti-PD-1-induced CD8^+^ T cell reinvigoration or CD8^+^ T cell activation markers, it increased tumor infiltration by CD8^+^CD3^+^ T cells, CD3^+^CD8^−^ helper T cells (CD4^+^), CD3^−^CD8^+^ plasmacytoid dendritic (pDC) cells, and CD45^+^ immune cells compared to anti-PD-1 alone (Fig. 2 *D*–*G* and *SI Appendix,* Fig. S2 *I*–*K*). These results show that the main effect of losartan on anti-HCC immunity was to increase tumor infiltration with reinvigorated T cells and pDC. Losartan cotreatment, however, did not enhance the anti-PD-1-induced expression of MHC-I related genes, which present antigens to CD8^+^ T cells (39), such as *Nlrc5*, *Psm9*, and *Tap1,* neither did it affect *Cd274* or *Il1b* messenger RNA (mRNA) expression (*SI Appendix,* Fig. S2 *L–P*).

### Losartan Inhibits Liver Fibrosis.

**Fig. 3.**
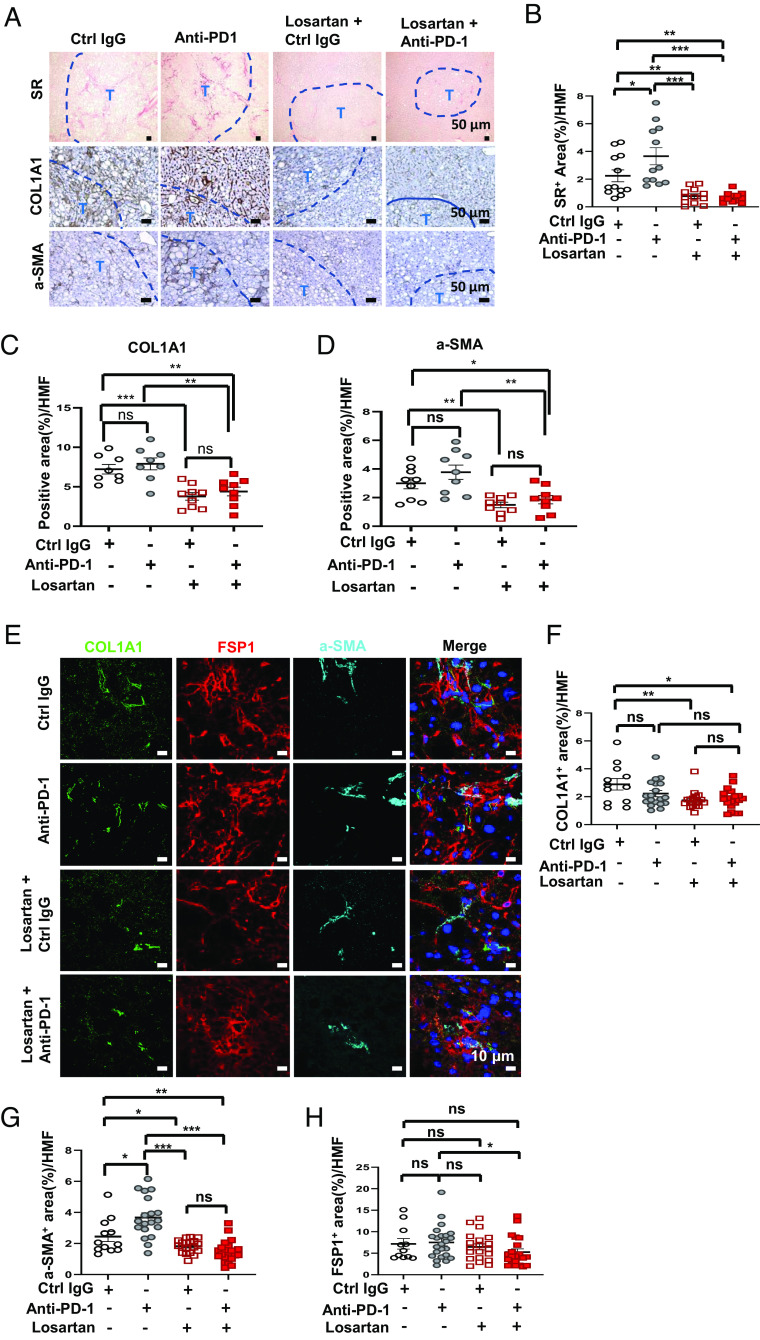
Losartan inhibits NASH-related liver fibrosis. (*A*) Formalin-fixed paraffin-embedded (FFPE) liver sections were stained with Sirius Red (SR) (*Top*), collagen I alpha 1 chain (COL1A1) (*Middle*), and α-SMA (*Bottom*) antibodies. (Scale bars, 50 µm) T, tumor. (*B–D*) SR (*B*), COL1A1 (*C*), and α-SMA (*D*) staining intensities per HMF were determined by Image J quantitation of 10 fields per section. (*E*) Frozen liver sections were stained for COL1A1, FSP1, and α-SMA and examined by fluorescence microscopy. (Scale bars, 10 µm.) Experiments were repeated at least three times. (*F–H*) Quantification of COL1A1^+^ (*F*), α-SMA^+^ (*G*), and FSP1^+^ (*H*) areas per HMF. Data are presented as mean ± SEM. *P < 0.05, **P < 0.01, ***P < 0.001, ns, not significant (unpaired two-tailed *t* test and Mann–Whitney *U* test).

We next explored likely mechanisms through which losartan stimulates HCC infiltration by T cells. Losartan alone or together with anti-PD-1 largely reduced liver fibrosis, assessed by Sirius Red staining, which was slightly increased by anti-PD-1 alone (Fig. 3 *A* and *B*). The major extracellular matrix (ECM) protein collagen type I a1 chain (Col1a1) and the activated HSC marker α-SMA also declined after losartan alone or losartan+anti-PD-1 (Fig. 3 *A* and *C–G*). Losartan, however, had a modest effect on fibroblast-specific protein 1 (FSP1)-expressing fibroblasts (Fig. 3 *E* and *H*). Unlike α-SMA^+^ HSC, FSP1^+^ fibroblasts support the response to immunotherapy by producing chemokines (40). Of note, losartan cotreatment increased the number of lymphoid-dense areas (tertiary lymphoid follicle-like structures), which contained FSP1^+^ fibroblasts next to B220^+^ B cells and CD8^+^ T cells (*SI Appendix,* Fig. S3 *A*–*C*) and shown to predict better prognosis (41). COX2^+^α-SMA^+^ fibroblasts and COX2^+^FSP1^+^ fibroblasts, which have immunosuppressive properties (42), were lower after losartan treatment (*SI Appendix,* Fig. S3 *D–G*). Altogether, losartan treatment reduced liver fibrosis, inhibited Col1a1 deposition, and blunted the generation of immunosuppressive CAF.

### Losartan Inhibits TGF-β Signaling.

**Fig. 4.**
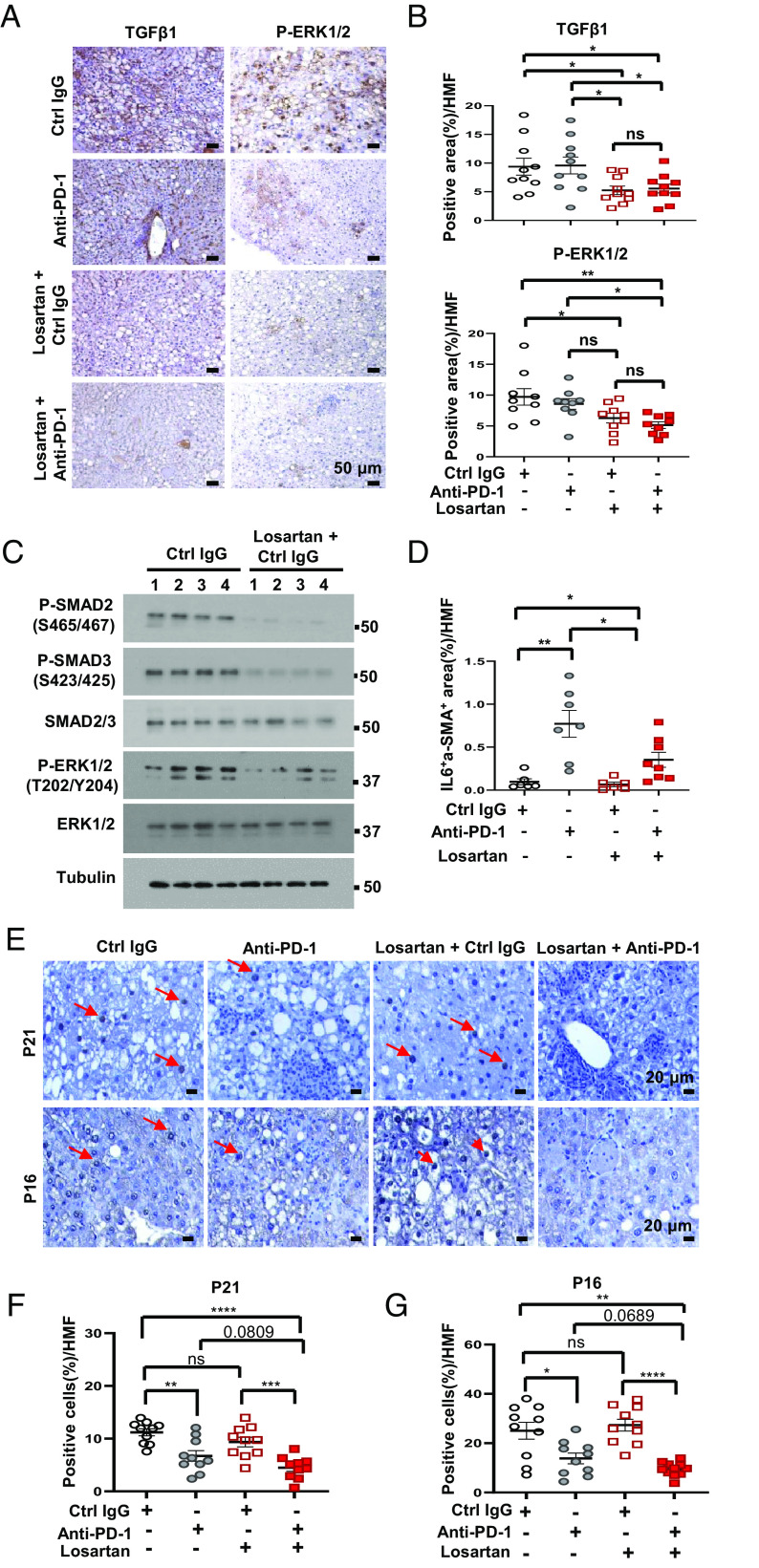
Losartan promotes stromal remodeling by inhibiting TGF-β expression and signaling. (*A*) IHC staining of TGF-β1 (*Left*) and phosphorylated (P)-ERK1/2 (*Right*) in FFPE liver sections from *MUP-uPA* mice in the different treatment groups. (Scale bars, 50 µm.) (*B*) TGF-β1 (*Top*) and P-ERK1/2 (*Bottom*) staining intensity per HMF determined by Image J. (*C*) Immunoblot analysis of the indicated proteins in liver lysates of mice from the indicated treatment groups. (*D*) Quantification of IL6^+^α-SMA^+^ area per HMF by Image J analysis of the data in *SI Appendix,* Fig. S4*E*. (*E–G*) IHC staining of p21 and p16 in FFPE liver sections from the indicated treatment groups (*E*). Scale bars, 20 µm. p21 (*F*)- and p16 (*G*)-positive cells per HMF determined by Image J. Data are presented as mean ± SEM. *P < 0.05, **P < 0.01, ***P < 0.001, ****P < 0.0001, ns, not significant (unpaired two-tailed *t* test and Mann–Whitney *U* test).

Next, we examined the effect of losartan on TGF-β signaling. IHC showed that losartan inhibited ERK1/2 phosphorylation in hepatocytes and stellate cells, as well as TGF-β1 expression (Fig. 4 *A* and *B*). Immunoblotting (IB) confirmed the decrease in ERK1/2 phosphorylation and showed that losartan also inhibited SMAD2 and 3 phosphorylation (Fig. 4*C*). Quantitative Real-Time PCR (qRT-PCR) showed that losartan also blunted *Tgfbr1*, *Tgfbr2*, *Vegf*, *Pdgfb*, *Pdgfrα*, *Pdgfrβ*, *Ctnnb1* (β-Catenin), and *Fgf2* mRNA expression (*SI Appendix,* Fig. S4 *A* and *B*). Expression of the TGF-β target connective tissue growth factor (CTGF) also decreased after losartan without or with anti-PD-1 (*SI Appendix,* Fig. S4 *A*, *C*, and *D*). In agreement with a recent publication, anti-PD-1 treatment increased IL-6 expression (Fig. 4*D* and *SI Appendix,* Fig. S4*E*), which supports ICI resistance (43). Losartan cotreatment, however, reversed this effect and reduced the number of IL6^+^ α-SMA^+^ fibroblasts (Fig. 4*D* and *SI Appendix,* Fig. S4*E*). Consistent with the ability of PD-1 blockade to improve senescence surveillance (44), anti-PD-1 treatment reduced p21 and p16 expression, an effect that was modestly enhanced by losartan cotreatment (Fig. 4 *E*–*G*). Anti-PD-1 increased the expression of hexosamine pathway (HBP) genes (*SI Appendix,* Fig. S4 *F–J*), including glutamine-fructose-6-phosphate transaminase 1 (*Gfpt1*), O-linked N-acetylglucosamine (*GlcNAc*) transferase (*Ogt*), phosphoglucomutase 3 (*Pgm3*), UDP-N-acetylglucosamine pyrophosphorylase 1 (*Uap1*), and the EGFR ligand amphiregulin (*Areg*), which promotes ECM hyaluronan synthesis (45). The addition of losartan, however, reduced the expression of these genes (*SI Appendix,* Fig. S4 *F–J*). Collectively, these results demonstrate that losartan has a profound effect on the ECM and the tumor stroma, effects which are consistent with the inhibition of TGF-β signaling and that are likely to contribute to enhancement of tumor invasion by CD8^+^ Teff cells.

## Discussion

Our results show that losartan, a safe, inexpensive, and widely used AngIIR1 antagonist, significantly potentiates HCC regression in response to PD-(L)1 blockade. Of note, losartan had no effect on the activation state of hepatic T cells and their expression of the inhibitory collagen receptor LAIR1, on its own or together with anti-PD-1. The only obvious effect of losartan cotreatment on anti-HCC immunity was the enhancement of HCC infiltration by CD8^+^ T cells that were reinvigorated by PD-1 blockade, as well as by pDC. Without added losartan, anti-PD-1 treatment resulted in the expected increase in Teff cells, but the reinvigorated CD8^+^ T cells mainly remained at the tumor margin with very few of them detected within the tumor proper. Consistent with previous publications (32, 33), losartan treatment alone or in combination with anti-PD-1 ameliorated liver and peritumoral fibrosis, an effect that was likely due to inhibition of TGFBR1, TGFBR2, and CTGF expression and SMAD and ERK phosphorylation, as well as diminished Col I production due to inhibition of HSC activation, all of which reflect the inhibition of TGF-β signaling. Indeed, the effects of losartan closely resemble the anti-PD-L1 potentiating effect of TGF-β1 blockade in a mouse model of colorectal cancer (46). These results are consistent with our previous finding that HCCs that were surrounded by more extensive peri-tumoral fibrosis were refractory to PD-L1 blockade compared to HCC nodules lacking a fibrotic envelope (19). Although it was already reported that losartan inhibits Col I production and improves blood vessel perfusion, leading to improved drug delivery and enhanced chemotherapy and radiation effectiveness (34, 47, 48), here losartan was shown to increase ICI responsiveness. Our results were obtained in a mouse model of NASH-driven HCC that shares many features with the equivalent human disease, including a highly similar transcriptome and mutational signature (17). Nonetheless, it is important to conduct retrograde analysis of human clinical data and determine whether HCC patients who received losartan or other AngIIR1 inhibitors exhibit an improved ORR when subjected to PD-(L)1 blockade. Moreover, it needs to be tested whether losartan can potentiate the response to PD-(L)1 blockade in other cancers associated with peritumoral fibrosis and desmoplasia, such as pancreatic cancer and intrahepatic cholangiocarcinoma, which so far have been refractory to ICI. As losartan only slightly affect hepatosteatosis, our results suggest that liver fibrosis maybe a more relevant explanation of the modest response of NASH-induced HCC to PD-(L)1 blockers (12–14).

## Materials and Methods

### Animals.

*MUP-uPA* mice were previously described and kindly provided by E.P. Sandgren, University of Wisconsin-Madison (49). The mice were maintained in filter-topped cages on autoclaved food and water with a 12 h light (6 am-6 pm)/dark (6 pm-6 am) cycle. To induce NASH and HCC, male mice were placed on HFD (Bio-Serv S3282) at 6 to 8 wk of age. After 6 mo, the mice were administered control IgG [25AUW, mouse [HEXON-Ad] mAb (TC31.27F11.C2) IgG1 D265A/Kappa] (20 mg/kg, i.p.) or anti-PD-1 [03AHF, mouse-modified PD-1 mAb (DX400 D265A LPD2127/LPD2128) mIgG1/Kappa], two to three times weekly (20 mg/kg, i.p.) without or with losartan (30 mg/kg in drinking water) (*SI Appendix,* Fig. S1*E*). For dose-finding studies, 30 mg/kg and 50 mg/kg losartan were used in drinking water, as indicted (*SI Appendix,* Fig. S1 *A–D*). The mice were monitored daily during treatment and were provided with soft bedding and nesting as well as access to food and water ad libitum. Body weight gain and food consumption were calculated every 2 wk. After 8 wk, the mice were killed, and tumors and livers were analyzed. Tumor volume was calculated as: L × W × H/2 (L, length, W, width, and H, height). All experiments were performed according to University of California San Diego (UCSD) Institutional Animal Care and Use Committee and NIH guidelines and regulations. Karin’s Animal protocol S00218 was approved by the UCSD Institutional Animal Care and Use Committee.

### FC.

Single**-**cell suspensions were prepared from livers and spleens. For liver lymphocyte isolation, 0.5 g of tissue was cut into small pieces and incubated in dissociation solution (DMEM medium supplemented with 5% FBS), collagenase type I (200 U/mL), collagenase type IV (200 U/mL), and DNase I (100 μg/mL) for 40 min at 37 °C. Next, the cell suspensions were passed through a 40-μm cell strainer and washed twice. The isolated cells were incubated with labeled antibodies in Cell Staining Buffer (BioLegend). Dead cells were excluded based on staining with Live/Dead Fixable Viability Dye (FVD-eFluor780, ThermoFisher Scientific/eBioscience). For intracellular cytokine staining, cells were restimulated with cell stimulation cocktail (ThermoFisher Scientific/eBioscience; containing PMA and ionomycin), in the presence of a protein transport inhibitor cocktail (ThermoFisher Scientific/eBioscience; containing brefeldin A and monensin). After 4-h incubation at 37 °C, cells were fixed and permeabilized with BD™ Perm/Wash buffer (BD Biosciences). After fixation/permeabilization, cells were stained with labeled antibodies of interest. The cells were analyzed on a Beckman Coulter Cyan ADP flow cytometer. Data were analyzed using FlowJo software (Treestar). Absolute numbers of specific immune cells (e.g., CD8^+^ cells) in spleens were calculated by multiplying the total cell numbers from one spleen by the percentage of the cell type in question among total CD45^+^ immune cells. Absolute immune cell numbers in livers were calculated by multiplying total cell number in one liver fragment by the percentages of the corresponding cell type among all total liver cells divided by the weight of the analyzed liver fragment (cell number per gram of liver). Antibodies were purchased from BD Biosciences, BioLegend, and ThermoFisher Scientific.

### Histology.

Livers were removed, and portions of liver tissue were fixed in 4% paraformaldehyde and embedded in paraffin. Thick sections (5 μm) were stained with hematoxylin and eosin (H&E) (Leica, 3801615, 3801571) and Sirius Red (ab246832) and processed for IHC. For frozen block preparations, liver tissue fragments were embedded in Tissue-Tek OCT compound (Sakura Finetek), sectioned, and stained with Oil Red O (ORO). Image J was used for image quantification as descried (19). Briefly, for Sirius Red, areas of at least 1 mm^3^ were quantitated with Image J and normalized for vascularization and lipid accumulation using corresponding H&E-stained areas. For ORO analysis, multiple images (3 to 4) were quantitated and averaged using Image J. IHC was performed as follows: after xylene deparaffinization and rehydration with ethanol series, antigen retrieval was conducted for 15 min at 100 °C with 0.1% sodium citrate buffer. After quenching of endogenous peroxidases with 3% H_2_O_2_ and blocking with 5% bovine serum albumin (BSA), sections were incubated with indicated antibodies (*SI Appendix,* Table S1) overnight at 4 °C followed by incubation with biotinylated secondary antibodies (1:200) for 30 min and Streptavidin-HRP (1:500) for 30 min. Bound peroxidase was visualized by 1 to 10 min incubation in 3, 30-diaminobenzidine (DAB) solution (Vector Laboratories, SK-4100). Images were captured on an upright light/fluorescent Image A2 microscope with AxioVision Release 4.5 Software (Zeiss).

### IB Analysis.

Livers were homogenized in a Dounce homogenizer (Thomas Scientific) with 30 strokes in RIPA buffer (50 mM Tris-HCl, pH 7.4, 150 mM NaCl, 1% Triton X-100, 1% sodium deoxycholate, 0.1% SDS, 1 mM Ethylenediaminetetraacetic acid (EDTA)) with complete protease and phosphatase inhibitor cocktail. Lysates were sonicated, centrifuged, and boiled in 4× loading buffer. The samples were separated by Sodium dodecyl-sulfate polyacrylamide gel electrophoresis (SDS-PAGE) and transferred to Polyvinylidene fluoride (PVDF) membranes, blocked in 5% nonfat milk, and incubated with the indicated primary antibodies overnight. Secondary antibodies were added for another 1 h. and detected with Clarity Western ECL Substrate (Biorad). Immunoreactive bands were exposed in an automatic X-ray film processor. Antibodies are listed in *SI Appendix,* Table S1.

### Immunostaining.

Tissues were embedded in Tissue Tek OCT (Sakura Finetek) and snap frozen. Tissue sections were fixed in cold acetone/methanol for 10 min and washed with Phosphate-buffered saline (PBS). Slides were blocked with PBS/1% normal donkey serum for 30 min. Sections were incubated with primary antibodies overnight at 4 °C. After washing with PBS, secondary antibodies and DAPI were added for 1 h at room temperature. Slides were washed with PBS and covered with FluorSave Reagent (EMD Millipore, 345789). Images were captured on a TCS SPE Leica confocal microscope. The results were quantified by counting dots/calculating intensity for each field of view (four to five areas for each slide) with Image J.

### Metabolic Measurements.

Liver and serum TG were measured with TG Colorimetric Assay Kit (Cayman Chemical #10010303) according to manufacturer’s protocol. Circulating ALT was measured with ALT(GPT) Reagent (Thermo Scientific™, TR71121) according to manufacturer’s protocol.

### RNA Isolation and qRT-PCR.

Total liver RNA was extracted with RNeasy Plus Mini kit (Qiagen #74134) and complementary DNA (cDNA) was synthesized with SuperScript™ VILO™ cDNA Synthesis Kit (ThermoFisher Scientific, 11754050). mRNA amounts were determined on a CFX96 thermal cycler (Biorad). Data were presented as arbitrary units and calculated by the comparative CT method [2Ct^(18s rRNA–gene of interest)^]. Primers are listed in *SI Appendix,* Table S2.

### Quantification and Statistical Analysis.

Data were presented as mean ± SEM. Differences between mean values were analyzed by two-tailed Student’s *t* test and Mann–Whitney *U* test with GraphPad Prism software. *P* value < 0.05 was considered as significant (*P < 0.05, **P < 0.01, ***P < 0.001, ****P < 0.0001).

## Supplementary Material

Appendix 01 (PDF)

## Data Availability

All study data are included in the article and/or *SI Appendix*.
