# Supplementary material for: Angiotensin II receptor inhibition ameliorates liver fibrosis and enhances hepatocellular carcinoma infiltration by effector T cells
Source: Proc Natl Acad Sci U S A. 2023 May 1;120(19):e2300706120. doi: 10.1073/pnas.2300706120 (PMC10175751; doi:10.1073/pnas.2300706120)
Supplement: Supplementary file 1 — Appendix 01 (PDF) [file pnas.2300706120.sapp.pdf]

Supplemental information for

Angiotensin II receptor inhibition ameliorates liver fibrosis and enhances hepatocellular carcinoma infiltration by effector T cells

Li Gu, Yahui Zhu, Maiya Lee, Albert Nguyen, Nicolas T Ryujin, Jian Yu Huang, Shusil K Pandit, Shadi Chamseddine, Lianchun Xiao, Yehia I. Mohamed, Ahmed O. Kaseb, Michael Karin*, Shabnam Shalapour*

*To whom correspondence may be addressed. Email: karinoffice@ucsd.edu, sshalapour@mdanderson.org

This PDF file contains:

Fig. S1-S4

Table S1-S2

Supplementary Figures

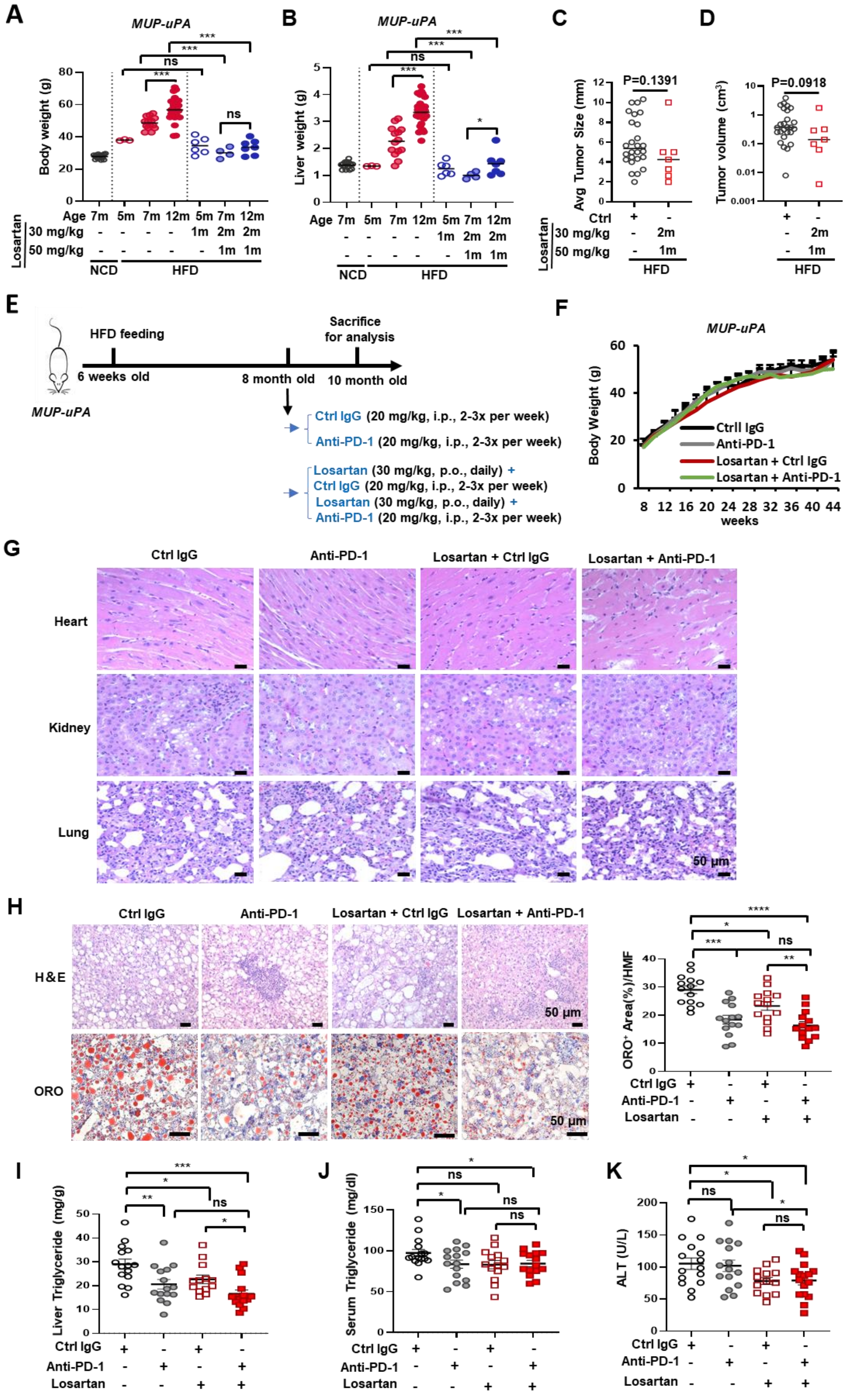

Fig. S1. Losartan potentiates anti-PD-1 induced HCC regression.

(*A and B*) Male *MUP-uPA* mice were fed high fat diet (HFD) and treated with losartan

for the indicated durations and dosage. body (A) and liver (B) weights at end point with Losartan in drinking water for 1 month at 30mg/kg or 3 months (2 months 30mg/kg+1 month 50mg/kg).

(*C and D*) Comparison of tumor size (C), and volume (D) at end point in 12 months HFD mice livers with indicated treatment.

(*E*) Outline of HFD-induced NASH-HCC in *MUP- uPA* mice and treatment scheme.

(*F*) Body weight gain by HFD-fed *MUP- uPA* mice subjected to the indicated treatments (n=13-15).

(*G*) H&E staining of heart, kidney, and lung tissues of mice from the different treatment groups outlined in S1E. Scale bars, 50 μm.

(*H*) H&E and Oil Red O (ORO) staining of liver sections from the different treatment groups (left). Scale bars, 50 μm. ORO staining intensity per high-magnification-field (HMF) determined by Image J (right).

(*I-K*) Liver (I) and serum (J) triglycerides (TG), and serum alanine aminotransferase (ALT) (K) in mice belonging to the different treatment groups.

Data are presented as mean ± SEM. *P < 0.05, **P < 0.01, ***P < 0.001, ****P < 0.0001; ns, not significant (Unpaired two-tailed t test and Mann-Whitney test).

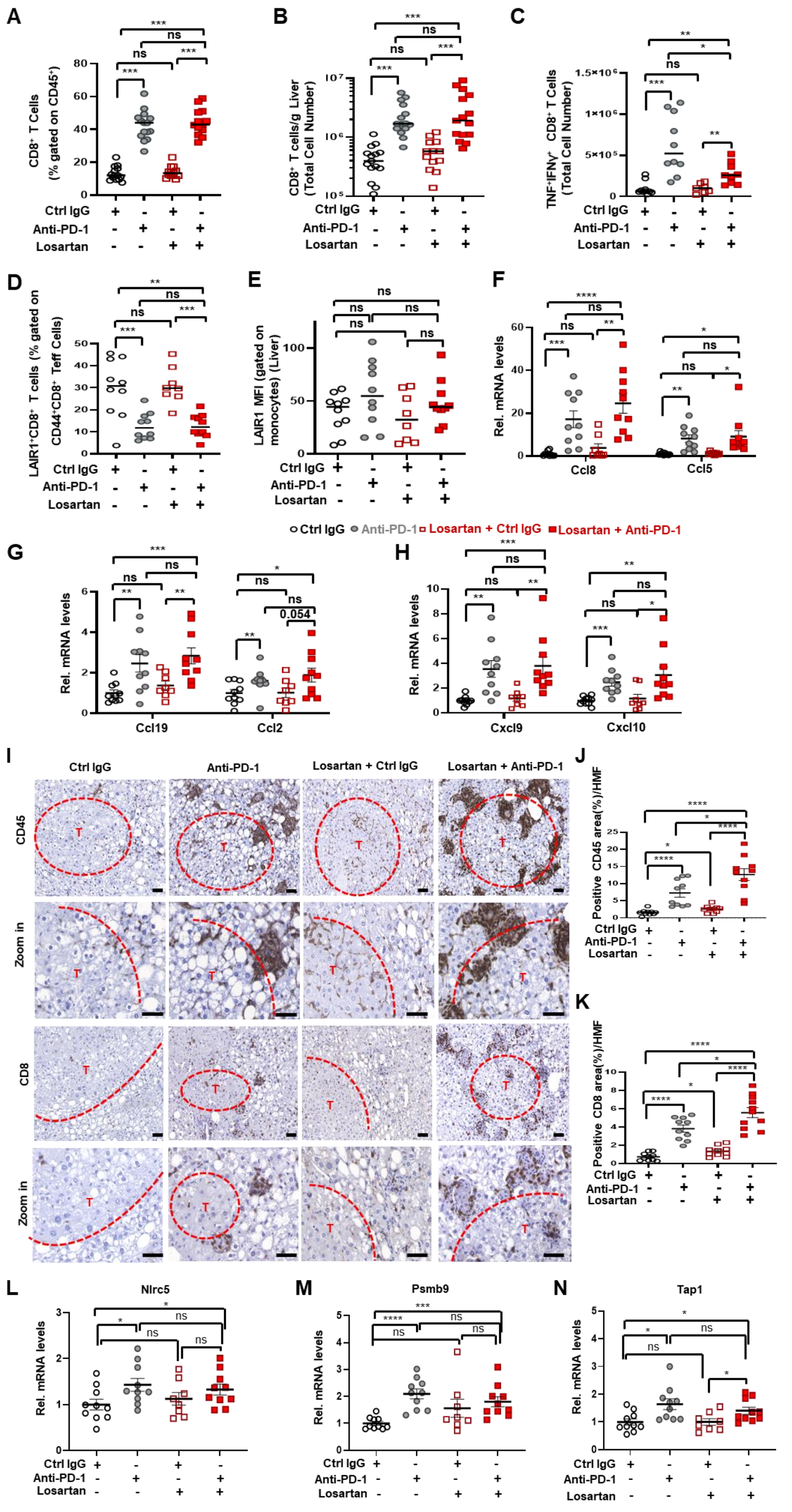

(Figure continued on next page)

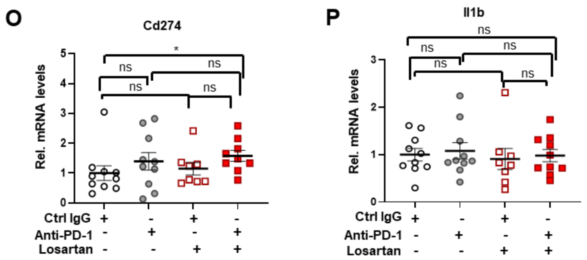

Fig. S2. Losartan stimulates intratumoral infiltration by anti-PD-1 reinvigorated Teff cells.

(*A*-*E*) Liver $CD8^+$ T cells as percentage of liver $CD45^+$ cells (A), $CD8^+$ T cell number/g liver (B), liver $TNF^+IFN\gamma^+$ $CD8^+$ T cell number (C), $LAIR1^+CD8^+$ cells as percentage of total liver $CD44^+CD8^+$ Teff cells (D) and Mean fluorescence intensity (MFI) of LAIR1 in monocytes from liver (E) determined by FC analysis of liver cell suspensions from the different treatment groups.

(*F*-*H*) Relative mRNA amounts of Ccl8, Ccl5 (F), Ccl19, Ccl2 (G), Cxcl9, and Cxcl10 (H) determined by Q-RT-PCR analysis of liver RNA from the indicated treatment groups.

(*I*) FFPE liver sections from the different treatment groups stained for CD45 and CD8. T-tumor, scale bars, 50 μm.

(*J* and *K*) Quantification of CD45 (J), and CD8 (K) positive areas per HMF determined by Image J.

(*L*-*P*) Q-RT-PCR quantitation of Nlrc5 (L), Psmb9 (M), Tap1 (N), Cd274 (O), and Il1b (P) mRNAs in livers from the indicated treatment groups.

Data are presented as mean ± SEM. $^*P < 0.05$, $^{**}P < 0.01$, $^{***}P < 0.001$, $^{****}P < 0.0001$; ns, not significant (Unpaired two-tailed t test and Mann-Whitney test).

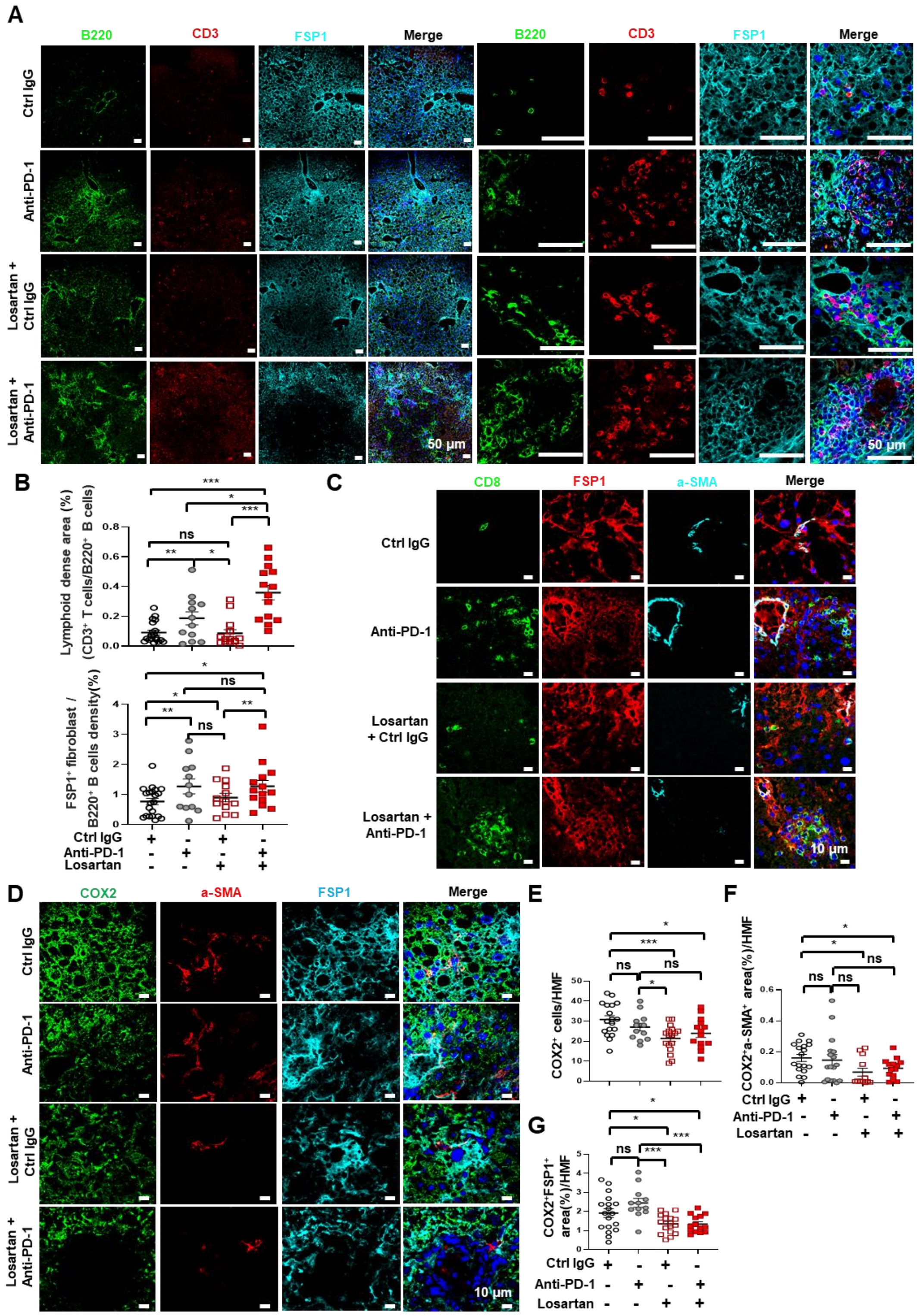

Fig. S3. Losartan decreases liver fibrosis induced by anti-PD-1 treatment.

(*A*) Frozen liver sections from the indicated treatment groups were stained for B220, CD3, and FSP1. Scale bars, 50 μm.

(*B*) Lymphoid dense areas (top) and $FSP1^+$ fibroblast/$B220^+$ B cell density (bottom) per HMF determined by Image J.

(*C*) Frozen liver sections from the indicated treatment groups were stained for CD8, FSP1, and α-SMA and examined by fluorescence microscopy. Scale bars, 10 μm.

(*D*) Frozen liver sections from the indicated treatment groups were stained for COX2, α-SMA, and FSP1. Scale bars, 10 μm.

(*E*-*G*) Quantification of areas occupied by $COX2^+$ (E), $COX2^+$α-SMA^+ (F), and $COX2^+FSP1^+$ (G) per HMF from the images shown in Fig. S3D.

Data are presented as mean ± SEM. *$P < 0.05$, **$P < 0.01$, ***$P < 0.001$; ns, not significant (Unpaired two-tailed t test and Mann-Whitney test).

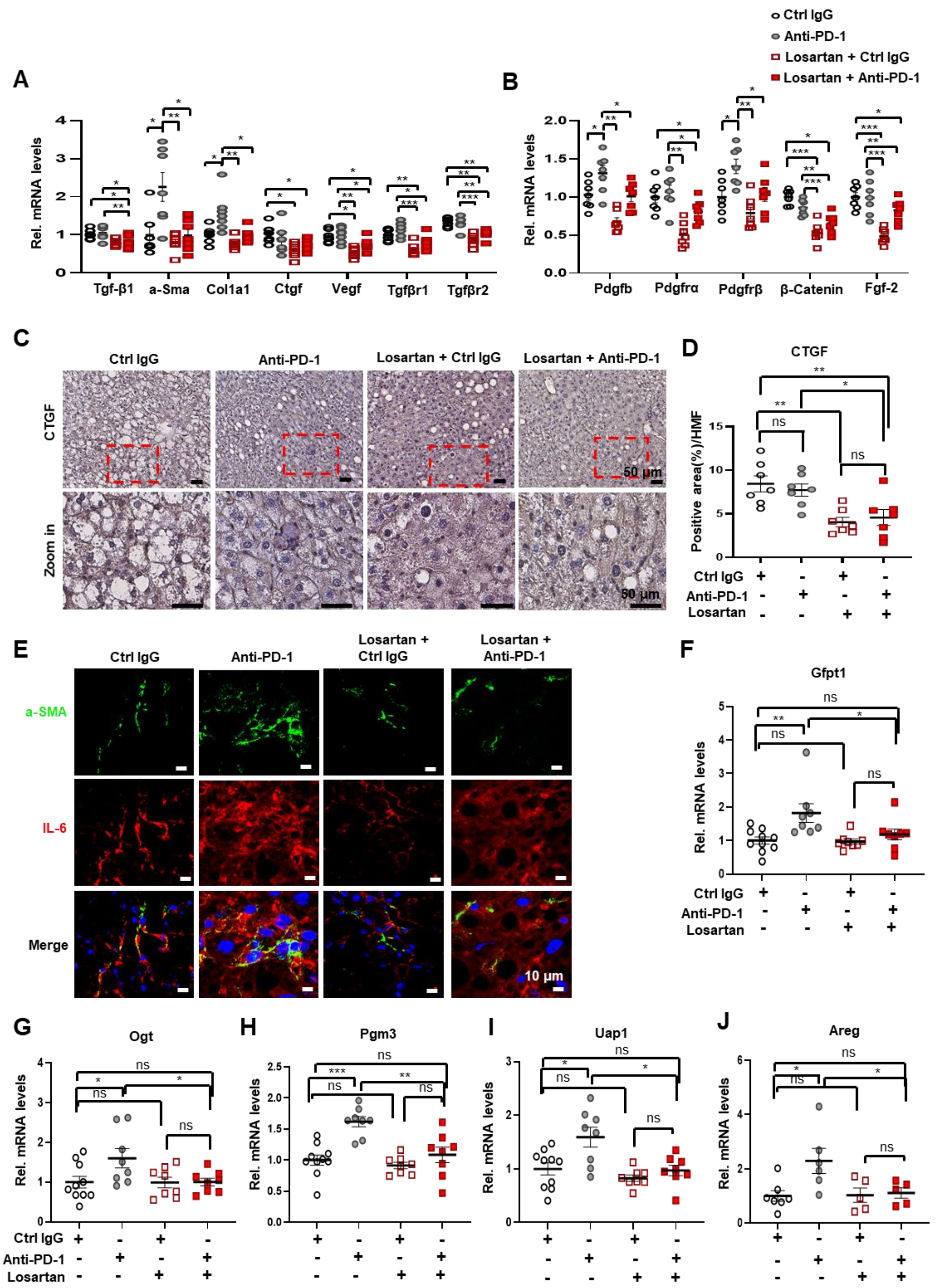

Fig. S4. Losartan promotes stroma remodeling, and represses the hexosamine biosynthetic pathway.

(*A* and *B*) Q-RT-PCR quantitation of mRNAs related to TGF-β (A) and PDGF (B) signaling in livers from the indicated treatment groups (n=7).

(*C* and *D*) FFPE liver sections from the different treatment groups were stained for CTGF (C), whose staining intensity (D) per HMF was determined by Image J. Scale bars, 50 µm.

(*E*) Frozen liver sections from the different treatment groups were stained for α-SMA and IL-6 and examined by fluorescence microscopy. Scale bars, 10 µm.

(*F-J*) Q-RT-PCR quantitation of mRNAs coding for the hexosamine biosynthetic pathway (F-I) and Areg (J) in livers from the indicated treatment groups (n=8-10).

Data are presented as mean ± SEM. *P < 0.05, **P < 0.01, ***P < 0.001; ns, not significant (Unpaired two-tailed t test and Mann-Whitney test).

Table S1. Antibodies used in this study.

Antibodies to:	Catalog number	Source	
p-SMAD2(S465/467)/ SMAD3(S423/425)	#8828	Cell Signaling	Rabbit
CD45	#70257	Cell Signaling	Rabbit
FSP1	ab197896	Abcam	Rabbit
CD3	IS503	Dako	Rabbit
COL1A1	72026	CST	Rabbit
COL1A1	sc8784	Santa Cruze	Goat
α-SMA	#19245	CST	Rabbit
TGFβ	SC130348	Santa Cruze	Mouse
P-ERK1/2	9101	Cell Signaling	Rabbit
P-SAMD2	18338	CST	Rabbit
P-SMAD3	9520	CST	Rabbit
SMAD2/3	8685	CST	Rabbit
ERK1/2	9102	CST	Rabbit
IL-6	sc-28343	Santa Cruze	Mouse
B220	13-0452-86	Thermo Fisher	Rat
COX2	PA1-9032	Thermo Fisher	Goat
P21	ab188224	Abcam	Rabbit
P16	sc-1661	Santa Cruz Biotechnology	Mouse
CD8	#98941	Cell Signaling	Rabbit

CD8	14-0081-85	Thermo Fisher	Rat
IFNγ	50-7311-82	Thermo Fisher	Rat
IL-17	25-7177-80	Thermo Fisher	Rat
CD4	48-0042-80	Thermo Fisher	Rat
CD19	50-0193-82	Thermo Fisher	Rat
IgA	11-4204-83	Thermo Fisher	Rat
CD8	45-0081-82	Thermo Fisher	Rat
TNF	11-7321-41	Thermo Fisher	Rat
B220	48-0452-82	Thermo Fisher	Rat
TIM3	134006	Biolegend	Rat
CD44	48-0441-82	Thermo Fisher	Rat
CD45	103137	Biolegend	Rat
PD-L1	25-5982-82	Thermo Fisher	Rat
CD138	142504	Biolegend	Rat
LAIR1	12-3051-82	Thermo Fisher	Armenian hamster

Table S2. Quantitative PCR primers used in this study.

mRNA	Forward Primer (5'-3')	Reverse Primer (5'-3')
Psmb9	GAAGAAGTCCACACCGGGAC	GAGGGGAGAGCTTGTCGAAC
Cd274	GCTCCAAAGGACTTGTACGTG	TGATCTGAAGGGCAGCATTTC

Nlrc5	GACGCTGGGGTTAACAGGAA	CAGCTCCACAAGACTCAGCA
Tap1	CCCAGCAGGTTCCATCACAT	GAAAAAGCAGGGGCAGGTTG
IL1b	GCCTCGTGCTGTCGGACC	TGTCGTTGCTTGGTTCTCCTTG
Ccl8	TCTACGCAGTGCTTCTTTGCC	AAGGGGGATCTTCAGCTTTAGTA
Ccl19	GGGGTGCTAATGATGCGGAA	CCTTAGTGTGGTGAACACAACA
Ccl5	GCTGCTTTGCCTACCTCTCC	TCGAGTGACAAACACGACTGC
Ccl2	TTAAAAACCTGGATCGGAACCAA	GCATTAGCTTCAGATTTACGGGT
Cxcl9	TGCCATGAAGTCCGCTGTTC	CTAGGGTTCCTCGAACTCCAC
Cxcl10	CCAAGTGCTGCCGTCATTTT	TTCATCGTGGCAATGATCTCAAC
α-Sma	CTGACAGAGGCACCACTGAA	GAAGGAATAGCCACGCTCAG
Tgf-β1	TTGCTTCAGCTCCACAGAGA	TGGTTGTAGAGGGCAAGGAC
Col1a1	GCTCCTCTTAGGGGCCACT	CCACGTCTCACCATTGGGG
Ctgf	CAAAGCAGCTGCAAATACCA	GTCTGGGCCAAATGTGTCTT
Tgf-βrI	GGTCTTGCCCATCTTCACAT	CAGGGGCCATGTACCTTTTA
Tgf-βrII	GCAAGTTTTGCGATGTGAGA	GGCATCTTCCAGAGTGAAGC
Vegf	CAAGATCCGCAGACGTGTAA	TTAATCGGTCTTTCCGGTGA
Pdgfrα	TGGCATGATGGTCGATTCTA	CGCTGAGGTGGTAGAAGGAG
Pdgfrβ	TCAACGACTCACCAGTGCTC	TTCACAGGCAGGTAGGTGCT
Pdgfb	TCCAGATCTCTCGGAACCTC	GGCTTCTTTCGCACAATCTC
β-Catenin	CTCTTCAGGACAGAGCCAATG	ATGCTCCATCATAGGGTCCA
Fgf-2	CCTTGCTATGAAGGAAGATGG	TCCGTGACCGGTAAGTATTG
Ogt	GACGCAACCAAACTTTGCAGT	TCAAGGGTGACAGCCTTTTCA

Pgm3	AGCAGTGGGATGCTATTTATGTC	TGTCTGCGCTTTCTTGTGAGT
Uap1	ACTCCCAGGGGCACTTCATTA	GGCCTCTGACTTTCCATTTGT
Areg	TCATGGCGAATGCAGATACA	GCTACTACTGCAATCTTGGA
Gfpt1	GAAGCCAACGCCTGCAAAATC	CCAACGGGTATGAGCTATTCC
